# Acute pancreatitis as an atypical manifestation of COVID-19: A report of 2 cases

**DOI:** 10.1016/j.amsu.2021.102693

**Published:** 2021-08-05

**Authors:** Samia Berrichi, Zakaria Bouayed, Khaoula Jebar, Ikram Zaid, Siham Nasri, Houssam Bkiyar, Imane Skiker, Brahim Housni

**Affiliations:** a Department of Intensive Care Unit, Mohammed VI University Hospital, Oujda, Morocco; b Radiology Department, Mohammed VI University Hospital, Oujda, Morocco; c Faculty of Medicine and Pharmacy, Mohammed 1st University, Oujda, Morocco; d Medical Simulation Training Center, Faculty of medicine and pharmacy, Oujda, Morocco

**Keywords:** Pancreatitis, COVID-19, Cytokine storm

## Abstract

**Introduction:**

Respiratory signs are the main revealing symptoms of the COVID-19 infection, however extra respiratory symptoms might as well occur, including digestive manifestations.

**Case report:**

In this paper, we report two cases of acute pancreatitis at the front line of the patient's symptomatology revealing a COVID-19 infection. Both patients had respiratory symptoms suggestive of COVID-19 and abdominal symptoms consistent with acute pancreatitis later-on confirmed through laboratory and CT findings. Our conservative management led to an improvement of the pancreatitis, though the first patient suffered from a severe form of COVID-19 justifying the using of mechanical ventilation and ECMO, while the second patient exhibited a milder form of COVID-19. Although both patients improved in terms of pancreatitis, the overall evolution was very different due to the extent of the respiratory involvement of COVID-19, as one patient exhibited a spectacular improvement of her respiratory state leading to a full recovery, the other patient suffered a rapid worsening of her acute respiratory distress leading to death following ECMO complications. Our two cases join only few cases of COVID-19-induced pancreatitis that have been reported in the literature.

**Discussion:**

in our discussion we highlight the association of COVID-19 and acute pancreatitis as it has been reported throughout literature, we then dive into the suggested physiopathological mechanisms that lay grounds for that association, before discussing our two cases, and emphasizing on the need of further studies to fully apprehend the scale of COVID-19's extra-pulmonary involvement in general, and pancreatic in particular.

**Conclusion:**

Acute pancreatitis is a sever condition involving potentially severe complications, COVID-19 is an emergent rare etiology recently identified as a causality.

## Highlights

• Extra respiratory manifestations of COVID including digestive manifestations have been reported more and more recently throughout literature.
• The expression of Angiotensin-Converting Enzyme 2 (ACE-2) in the pancreatic glands renders the pancreas a potential target for the virus.
• The causal relationship between COVID-19 and Acute pancreatitis and its impact on the outcome is yet to be fully apprehended.

## 1 Introduction

COVID-19 is typically known to affect the respiratory system causing a variety of symptoms from a common cold-like presentation to a severe acute respiratory syndrome (SARS) hence the name SARS-Cov-2.

With over a 146 million cases listed globally to this day [1] and thousands of cases being reported every day, it has become clear that COVID-19 is more of a systemic disease that can manifest in multiple forms.

We report the cases of two patients hospitalized in our department for the management of Acute pancreatitis due to a COVID-19 infection.

## 2 Case report

### 2.1 Case 1

A 36-year-old women, with a history of a cholecystectomy 6 years ago was admitted to a local hospital for the management of COVID-19 symptoms made of cough, shortness of breath, and headaches with a positive SARS-Cov-2 RT-PCR. Within a week the patient's dyspnea worsened and she presented a sharp epigastric pain. She was then transferred to our department for management.

**Fig. 1:**
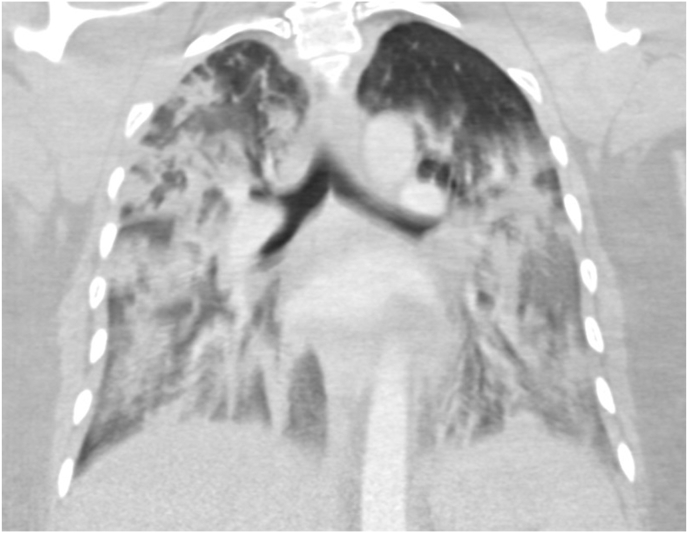
Coronal lung window showing central and peripheral ground-glass opacities in both lungs with interlobular septal thickening realizing a crazy paving pattern, bilateral pulmonary consolidation.

**Fig. 2:**
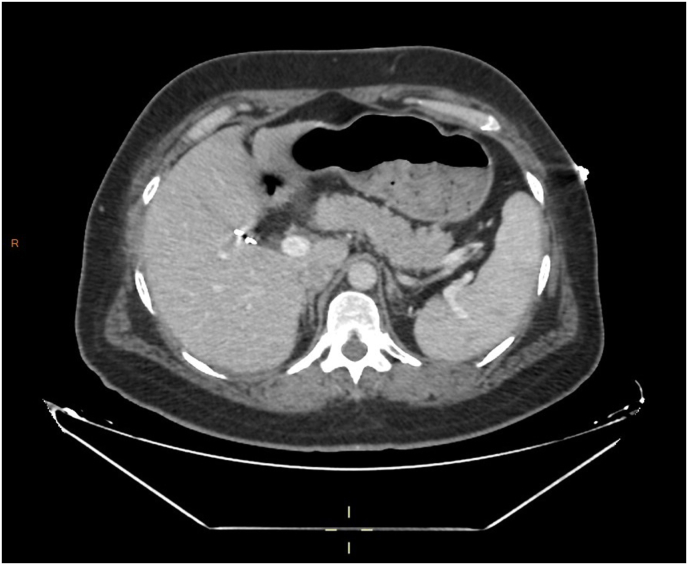
Axial C+ portal venous phase showing a swollen pancreas, enhanced uniformly, with discrete edema in the peri-pancreatic fat. Balthazar C pancreatitis without necrosis.

On physical examination, the patient had a BMI of 30.82, she was afebrile (37.8 °C), tachycardic (103 bpm) with an oxygen saturation of 36 % on ambient air and 67 % under High Flow Therapy (100 % FiO2 at a 60L/min flow rate) with signs of respiratory distress (labored breathing, suprasternal retractions, and paradoxical breathing), abdominal examination showed a mild epigastric tenderness. Laboratory findings showed an elevated WBC (16,68 × 10 [3]/μL), a high serum lipase levels at 2570 U/L, a CRP level of 289,80 mg/L, with a hemoglobin at 12,4 g/dl, a serum ferritin level of 542,72 ng/mL, an IL-6 level of 24pg/Ml and a normal electrolyte levels, as well as normal liver and kidney function tests (Table 1). Chest CT showed central and peripheral ground-glass opacities in both lungs with interlobular septal thickening realizing a crazy paving pattern, as well as a pulmonary consolidation predominant in the lower lobes, abnormalities that are consistent with a COVID-19 pneumonia and involving overall more than 75 % of the lungs (Fig. 1). Abdominal CT showed a diffuse enlargement of the pancreas with a discrete infiltration of the peripancreatic fat with no pancreatic necrosis consistent with a CTSI's C grade (Fig. 2).

**Table 1:** Laboratory findings.

	CASE 1	CASE 2
WBC (10^3^/ΜL)	16,68	14,13
LYMPHOCYTES (10^3^/ΜL)	220	1910
SERUM LIPASE (U/L)	2570	676
LDH (UI/L)	3325	760
SERUM CREATININE (MG/L)	10.94	9.98
SERUM FIBRINOGEN (G/L)	2.9	6.7
D-DIMERS (NG/ML)	810	2970
CRP (MG/L)	289,80	190.58
SERUM FERRITIN (ΜG/L)	542.72	570.51

The patient was treated with Azythromycine, High doses of methylprednisolone, intravenous paracetamol, optimization of electrolyte balance and a 5 days course of plasmapheresis.

The rapid worsening of the respiratory function with a PaO2/FiO2 ratio reaching 29 justified the use of mechanical ventilation therapy and the establishment of a Bi-Femoral VV-ECMO (Veno-venous Extra-Corporeal Membrane Oxygenation) set at 6900 rpm pump flow, and 3,9 L/min O_2_ flow, with standard heparin for ECMO. The patient also benefited from an advanced hemodynamic monitoring using *VIGILEO*® (continuous monitoring of invasive blood pressure, cardiac index, Central venous oxygen saturation (ScvO2), and Systemic vascular resistance index (SVRI)).

Follow-up laboratory tests showed a decline in lipase levels down to 311 U/L.

22 days following admission the patient died from ECMO hemorrhagic complications.

### 2.2 Case 2

**Fig. 3:**
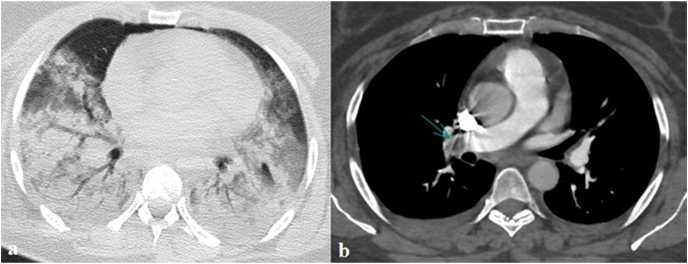
a: Axial lung window showing multiple confluent ground-glass opacities and consolidative areas predominantly in the lower lobes. SARS-Cov-2 RT-PCR positive, CT findings consistent with COVID-19 pneumonia CO-RADS 6. b: Axial C + CTPA showing a filling defect seen within the distal right main pulmonary artery (right proximal pulmonary embolism).

**Fig. 4:**
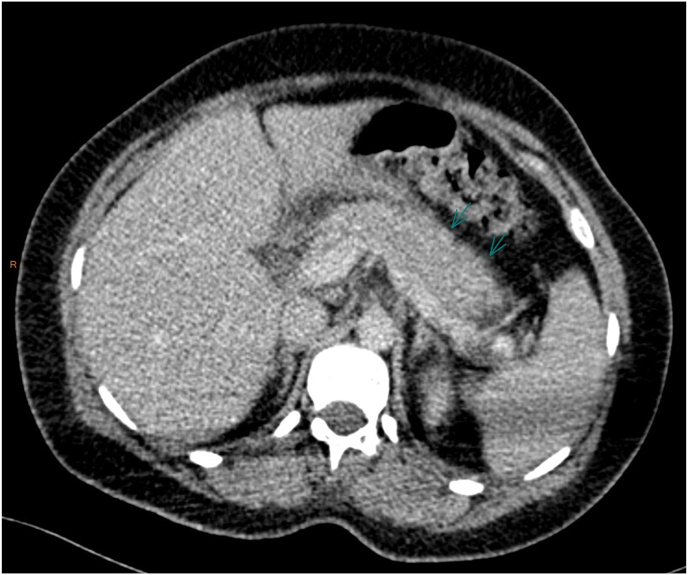
Axial C+ portal venous phase showing a mild swelling of the pancreas, uniformly enhanced. Balthazar B pancreatitis without peripancreatic abnormality or necrosis.

A 51-year-old women, with a history of a hyperthyroidism under Carbimazole, presented to our ER complaining of severe epigastric pain radiating to the back, nausea and vomiting, as well as shortness of breath. On physical examination, the patient had a BMI of 31,5, she was afebrile (37.5 °C), tachycardic (129 bpm) with an oxygen saturation of 79 % on ambient air and 84 % under 8L/min of O_2_, abdominal examination showed a sever epigastric tenderness. Laboratory findings showed an elevated WBC (14,13 × 10 [3]/μL), a high serum lipase levels at 676 U/L, a CRP level of 190,58 mg/L, with a hemoglobin at 11,9 g/dl, a serum ferritin level of 570,51 ng/mL (Table 1), as well as normal electrolyte levels, normal liver and kidney function tests, with a positive SARS-Cov-2 RT-PCR. Chest CT showed ground-glass opacities in both lungs predominantly in the lower lobes involving overall less than 10 % of the lungs, associated to a right proximal pulmonary embolism (Fig. 3). Abdominal CT showed a diffuse enlargement of the pancreas without any pancreatic or peripancreatic abnormalities, nor any collections or necrosis, consistent with a CTSI's B grade (Fig. 4).

The patient was treated with Azythromycine, dexamethasone, intravenous paracetamol, optimization of electrolyte balance and fluid therapy. She had a swift recovery and was discharged within 4 days with a total resolution of her respiratory and abdominal symptoms.

## 3 Discussion

The scope of the novel COVID-19 infection is yet to be fully determined. Initially defined as a viral pneumonia involving a wide range of clinical manifestations [2], the occurrence of thousands of cases daily and subsequent publishment of numerous studies has unequivocally proven that COVID-19 is more of a multi-systemic disease [3].

On the other hand, acute pancreatitis is an inflammation of the exocrine pancreas commonly caused by gallstones and alcohol consumption. Intrapancreatic protease activation damaging Acinar cells as well as recruitment of circulatory neutrophils being the underlying mechanism of acute pancreatitis [4].

The association of COVID-19 and Acute pancreatitis has been suggested in recent case reports [5,6]. Both Wang et al. [7] and Liu et al. [8] reported a 17 % incidence of pancreatic injury in 52 and 67 COVID-19 patients respectively.

A decisive link of causality between COVID-19 and Acute pancreatitis is yet to be proven [9]. The expression of Angiotensin-Converting Enzyme 2 (ACE-2) in both exocrine and endocrine pancreatic glands renders the pancreas a potential target for the virus [8], ACE-2 being the entry point for the SARS-Cov-2 initiating the COVID-19 pathogenesis [10,11] Though the pancreatic injury likely involves both a direct and an inflammatory-mediated process.

We describe the cases of two women both exhibiting symptoms of a COVID-19 pneumonia and gastro-intestinal symptoms suggestive of acute pancreatitis, later-on confirmed through laboratory and CT findings. Both patients’ digestive symptoms improved and serum lipase levels decreased thus evading the need of any medication or intervention regarding potential pancreatitis-related complications. Thought the first patient respiratory function rapidly worsened leading to death following ECMO complications, while the second patient evolved favorably.

The causal relationship between COVID-19 and Acute pancreatitis, more importantly its impact on the prognosis of patients suffering such association, is yet to be fully apprehended. Overall, it underlines the complex pathogenesis of COVID-19 and its extra-pulmonary injuries [12] as well as its implications in terms of management and prognosis.

## 4 Conclusion

Since fist identified, COVID-19 has rapidly spread and has proven to be a multi-systemic disease. Our case report highlights the importance of considering and addressing extra-pulmonary injuries in COVID-19 patients.

Further work is needed to establish a definitive causality between the SARS-Cov-2 virus and acute pancreatitis.

This work has been reported in line with the SCARE 2020 Guidelines [13].

## Ethical approval

The ethical committee approval was not required given the article type (case report).

## Sources of funding

This research did not receive any specific grant from funding agencies in the public, commercial, or not-for-profit sectors.

## Author contribution

BERRICHI SAMIA: Study conception, Data collection; data analysis; writing review & editing, BOUAYED ZAKARIA: Study conception, data analysis, JEBAR KHAOULA; ZAID IKRAM: Contributors, BKIYAR Houssam: Supervision and data validation, HOUSNI Brahim: supervision and data validation.

## Registration of research studies

As this manuscript was a case report with no new medical device nor surgical techniques, not prior registration is required.

## Guarantor

BERRICHI SAMIA.

BOUAYED M ZAKARIA.

## Consent

Written informed consent was obtained from the patient for publication of this case report and accompanying images.

## Declaration of competing interest

The authors state that they have no conflicts of interest for this report.
